# Loss of *GW5* function is involved in the unique grain shape of “Tanpo”, a Japanese landrace rice

**DOI:** 10.1270/jsbbs.24076

**Published:** 2025-03-27

**Authors:** Minami Ikeda, Ko Chiba, Ryouta Nakajima, Shinichi Matsumoto, Akio Watanabe, Kenji Ueda, Hiromori Akagi, Kenji Sakurai

**Affiliations:** 1 Department of Plant Production, Faculty of Bioresources, Akita Prefectural University, Kaidoubata-Nishi 241-438, Shimoshinjyo-Nakano, Akita 010-0195, Japan; 2 Agriculture, Forestry and Fisheries Promotion Department Extension Office, Niigata Prefecture, Nakaoki 684, Sado, Niigata 952-1211, Japan; 3 Akita Prefectural Agricultural Experiment Station, Genpachizawa 34-1, Aikawa, Yuwa, Akita 010-1231, Japan

**Keywords:** landrace, *GW5*, rice, grain shape, brassinosteroid

## Abstract

“Tanpo”, a Japanese rice landrace widely cultivated approximately 120 years ago in Akita Prefecture, exhibits a shorter, wider, thicker, and heavier grain compared to Akitakomachi. Microscopic analysis has revealed that the epidermal cells of Tanpo spikelet hulls are narrower and shorter, with an increased number of cells in the grain width direction, thus resulting in a distinctive grain shape. In a genetic analysis of an F_2_ population derived from a cross between Tanpo and Akitakomachi, the Tanpo *GW5* allele was found to determine the grain shape in a recessive manner. The *GW5* allele in Tanpo is a loss-of-function allele because it generates a stop codon immediately after the start codon with a 100-bp deletion within the first exon. Because the GW5 protein suppresses glycogen synthase kinase 2 (GSK2), a negative regulator of brassinosteroid (BR) signaling, GW5 deficiency in Tanpo results in reduced BR signaling. As a result, the expansion of epidermal cells was suppressed, while the radial cell division was promoted, which led to thicker and shorter spikelet hulls and, ultimately, the characteristic grain shape of Tanpo. The identification of this unique allele in the Tanpo landrace provides a valuable resource for breeding new rice varieties with unique grain characteristics.

## Introduction

Rice landraces are genetically diverse and exhibit a large variation in morphology (Hour *et al.* 2020, Kobayashi *et al.* 2006). These landraces possess unique characteristics and alleles that have been lost in modern cultivars; thus, they are valuable genetic resources for breeding (Jackson 1997, Yamaguchi *et al.* 2007). In Japan, rice landraces have adapted to the specific climatic conditions of each region and were cultivated until approximately 120 years ago.

Various landraces have been documented in Akita Prefecture, northern Japan, including “Tanpo”, which was widely cultivated in the northern region (Akimoto 1995, Saito 2006). We cultivated the Tanpo landrace, which had been preserved in the Genebank, using modern rice cultivation techniques and observed that the harvested brown rice grains were short and round in shape (Supplemental Fig. 1).

Numerous quantitative trait loci (QTLs) associated with the rice grain shape have been identified (Huang *et al.* 2013), and several genes encoding these QTLs have been isolated (Duan *et al.* 2017, Fan *et al.* 2006, Ishimaru *et al.* 2013, Li *et al.* 2011, Liu *et al.* 2017, Song *et al.* 2007, Wang *et al.* 2012). These genes regulate grain shape via networks of phytohormone processes and multiple signaling pathways (Chen *et al.* 2021, Li *et al.* 2022).

*GW5* (*qSW5*) has been identified as a major QTL responsible for the differences in grain width and weight between Japonica and Indica rice (Shomura *et al.* 2008, Wan *et al.* 2008, Weng *et al.* 2008), and it has been shown to encode a calmodulin-binding protein (Duan *et al.* 2017, Liu *et al.* 2017). A 1212-bp deletion in the promoter region of Japonica rice reduces the expression of *GW5*, which results in increased grain width (Duan *et al.* 2017, Liu *et al.* 2017). Furthermore, the GW5 protein, localized in the plasma membrane, regulates brassinosteroid (BR) signaling by suppressing glycogen synthase kinase 2 (GSK2), a negative regulator of BR signaling, thereby determining the grain shape (Liu *et al.* 2017).

In this study, we investigated the role of the *GW5* gene in the characteristic grain shape of the Tanpo landrace and proposed a mechanism by which the Tanpo *GW5* allele influences the grain shape.

## Materials and Methods

### Plant material

We used the Japanese landrace Tanpo (JP7283, maintained at the NARO Gene Bank), the Japanese cultivar Akitakomachi, and their F_2_ population. Forty-two of each of the Tanpo and Akitakomachi cultivars, along with 116 individuals from the F_2_ population, were transplanted into an experimental paddy field at the Akita Agricultural Experiment Station on May 30, 2023, with a planting density of 20.7 plants m^–2^. Fertilizer was applied before planting, which consisted of: N, 0.6 kg a^–1^; P_2_O_5_, 0.6 kg a^–1^; and K_2_O, 0.6 kg a^–1^. No additional fertilizer was provided.

### Morphological analyses of the rice grains

Mature rice plants were harvested on October 1, 2023, and dried in a well-aerated room for one month. Brown rice grains with a thickness of ≥1.9 mm were selected using a 1.9-mm sieve (Fuji Kinzoku Co., Ltd.). Approximately 200 grains from each sample were measured for grain length, width, and thickness using a Rice Analyzer (RGQI 20A; Satake Co., Ltd.). The moisture content of the rice grains was determined using a digital moisture meter (PB-1D2; Kett Electric Laboratory Co., Ltd.), and then the grain weight was converted to 15% moisture content.

### Electron microscope analysis of the epidermal cells of spikelet hulls

Spikelet hulls of the Tanpo and Akitakomachi cultivars were sampled two weeks after heading for electron microscope analysis. Four spikelet hulls of average size were selected, which were then coated with a multi-coater VES-10 (Vacuum Devices) and observed for epidermal cell morphology using the Miniscope TM3030Plus (Hitachi High-Tech Corporation). The length and width of the surface cells were measured from the photographed images.

### DNA extraction

Leaf tips from both parental varieties and F_2_ individuals were sampled 28 days after transplanting and stored at –20°C. Extraction buffer (0.1 M Tris-HCl [pH 8.0], 10 mM EDTA [pH 8.0], and 1 M KCl) was added to each sample with a stainless-steel ball (φ3 mm), and DNA was extracted using a multi-bead shocker (MB1021; Yasui Kikai Corporation) at 1600 rpm for 40 s. DNA was precipitated from 100 μL of the supernatant by adding an equal volume of 2-propanol. The DNA pellet was washed with 70% ethanol, air-dried, and suspended in 50 μL of 1/10 TE. The extracted DNA was used for PCR analysis.

### PCR

PCR was performed using *Premier EX* (Takara Bio Inc.) with the primer pairs that are listed in Supplemental Tables 1 and 2. The PCR conditions were an initial denaturation of 1 min at 94°C, followed by 35 cycles of 10 s at 98°C, 15 s at 55°C, and 1 min at 68°C, using a PCR9700 thermocycler (Thermo Fisher Scientific Inc.). Polymorphisms in the PCR products were detected by electrophoresis using 1% UltraPure agarose (Thermo Fisher Scientific Inc.) in 1× TAE buffer (Supplemental Tables 1, 2).

### Sequencing of the *GW5* gene

The entire region of the *GW5* gene was amplified from the Tanpo and Akitakomachi cultivars by PCR using the GW5-F1 and GW5-R4 primers (Supplemental Table 2). The amplified DNA fragments were purified using a QIAquick PCR Purification Kit (QIAGEN), followed by sequencing using an ABI PRISM^®^ 3100 Genetic Analyzer (Thermo Fisher Scientific Inc.). The nucleotide sequence data for the Tanpo GW5 gene has been deposited in the DDBJ database under the accession number LC848690.

### RNA extraction and RT-qPCR

Six young inflorescences (approximately 1 cm in length) were sampled from each of the Tanpo and Akitakomachi cultivars and stored at –80°C. Total RNA was extracted using the TRIzol^TM^ Plus RNA Purification Kit (Invitrogen), and RNA concentration was measured using a NanoDrop^TM^ One spectrophotometer (Thermo Fisher Scientific Inc.). Complementary DNA was synthesized from 50 ng of total RNA using a ReverTra Ace qPCR RT Master Mix with gDNA Remover (TOYOBO). RT-qPCR was performed using a THUNDERBIRD Next SYBR qPCR Mix (TOYOBO) with the primers that are listed in Supplemental Table 3 (Liu *et al.* 2017). Real-time PCR was performed with an initial denaturation at 95°C for 30 s, followed by 40 cycles of 5 s at 95°C and 30 s at 60°C for 30 s, using a Bio-Rad CFX96 Real-Time System (Bio-Rad). Melting curve analysis was performed from 65°C to 95°C. Data analysis was performed using Bio-Rad CFX Manager 3.1 (Bio-Rad), and the relative gene expression levels were calculated relative to the ubiquitin gene (Os03g0234200) using the 2-^ΔΔ^Ct method.

### Statistical analysis

Differences between the Tanpo and Akitakomachi cultivars were evaluated using the Student’s *t*-test with the data analysis module in Microsoft Office Excel 2019. Furthermore, differences in grain length, width, thickness, and weight among the *GW5* genotypes of the F_2_ population were determined by Tukey’s test using the R package 4.0.0. Statistical significance was established at *P* < 0.05.

## Results

### Features of Tanpo grains and spikelet hulls

**Fig. 1.**
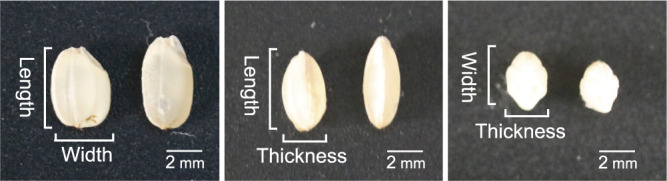
Comparison of the grain shape between the Tanpo (left) and Akitakomachi (right) cultivars from three angles. Scale bar = 2 mm.

The grains of Tanpo were shorter and wider than those of Akitakomachi (Supplemental Fig. 1). The grain length of Akitakomachi was 5.38 mm, whereas that of Tanpo was significantly shorter at 4.86 mm. Conversely, Tanpo grains were significantly wider (2.92 mm) and thicker (2.21 mm) than the Akitakomachi grains (2.77 mm and 2.07 mm, respectively). Similarly, the grain weight was also significantly higher in Tanpo (26.32 mg) compared to Akitakomachi (23.04 mg) (Fig. 1, Table 1). Thus, Tanpo grains were shorter, wider, thicker, and heavier than those of Akitakomachi.

**Fig. 2.**
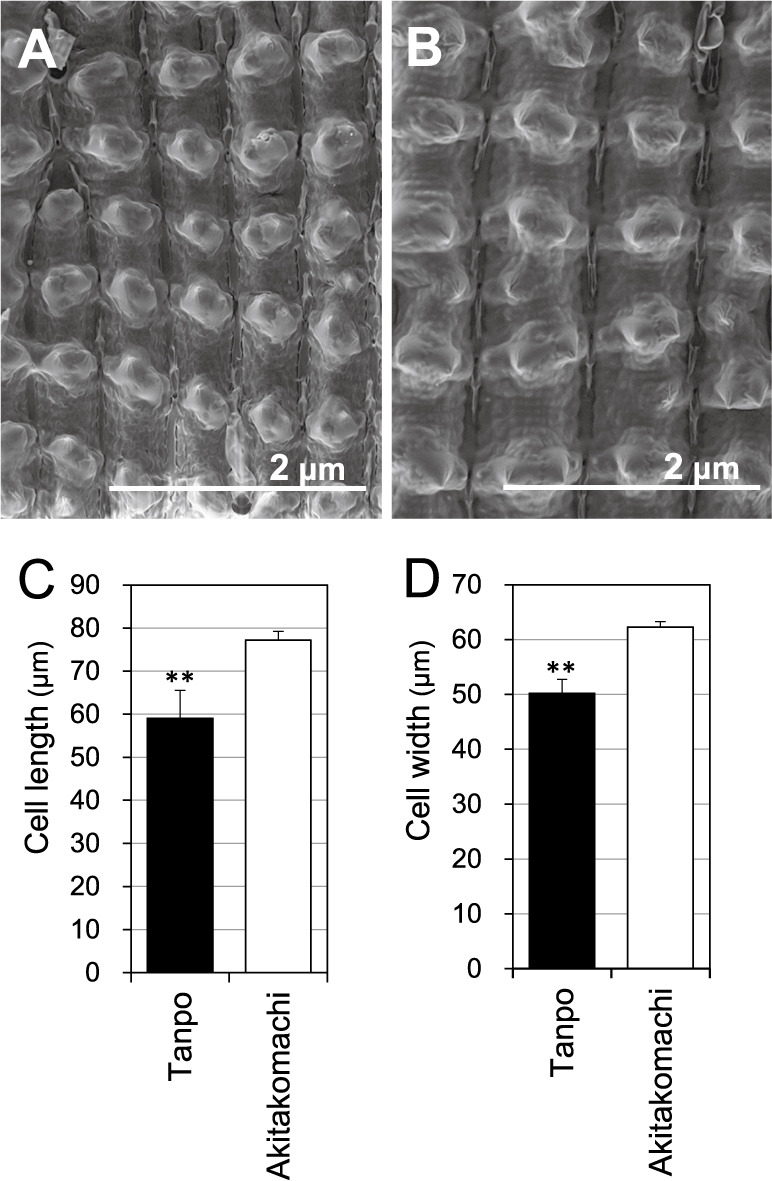
Comparison of epidermal cells of the spikelet hulls of Tanpo and Akitakomachi. A (Tanpo) and B (Akitakomachi) show the scanning electron microscope analysis of the epidermal cells of the spikelet hulls after two weeks of heading. Scale bar = 2 μm. C and D show the cell width and cell length, respectively. Black and white bars represent the Tanpo and Akitakomachi cultivars, respectively. Values represent the mean ± standard deviation (SD) (n = 4). Double asterisks indicate significant differences between Tanpo and Akitakomachi as determined using the Student’s *t*-test (**P < 0.01).

Because rice grains develop within fully formed spikelet hulls, which constrain the grain shape, we analyzed the epidermal cells of the spikelet hulls using electron microscopy (Fig. 2A, 2B). Epidermal cells in Tanpo hulls were significantly shorter (59.0 μm) and narrower (50.2 μm) than those of Akitakomachi (77.2 μm and 62.3 μm, respectively) (Fig. 2C, 2D). Although Tanpo spikelet hulls were narrower than Akitakomachi hulls, they were wider, which suggests a greater number of epidermal cell rows in the Tanpo cultivar (Supplemental Fig. 2). Therefore, the distinctive grain shape of Tanpo is likely due to the combination of limited cell expansion and the increased number of cell rows in their spikelet hulls.

### Linkage analysis of the *GW5* gene and grain shape in the F_2_ population

The *GW5* gene has previously been demonstrated to control rice grain width and weight (Duan *et al.* 2017, Liu *et al.* 2017). To investigate whether the *GW5* gene was involved in the characteristic grain shape of Tanpo, we analyzed the linkage between the *GW5* genotype and grain traits in an F_2_ population between the Tanpo and Akitakomachi cultivars.

**Fig. 3.**
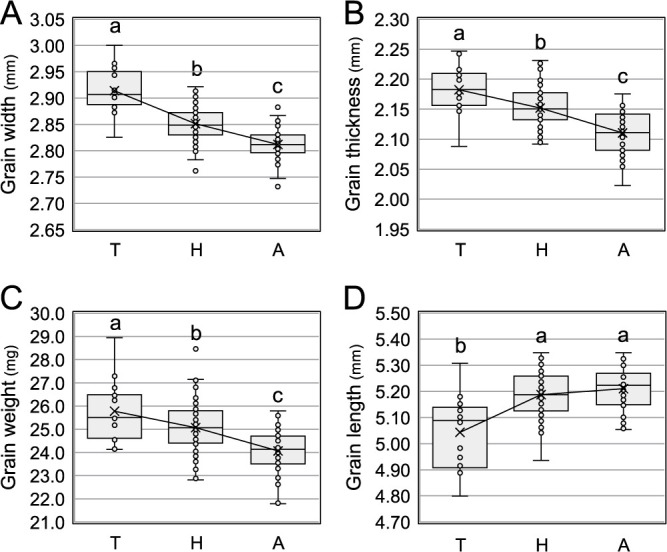
Box plot representing the grain characteristics among the *GW5* genotypes in F_2_ individuals of a cross between Tanpo and Akitakomachi. Panels A, B, C, and D show the grain width, thickness, length, and weight, respectively. The letters T, H, and A in each plot indicate homozygous for the Tanpo allele, heterozygous alleles, and homozygous for the Akitakomachi allele, respectively. The average of each box plot is indicated with an X and connected by a line. Box plots with different letters (a, b, and c) indicate statistically significant differences using the Tukey test (*P* < 0.05).

F_2_ individuals that were homozygous for the Tanpo *GW5* allele exhibited significantly wider, thicker, and heavier grains compared to those homozygous for the Akitakomachi allele, with intermediate phenotypes observed in heterozygotes (Fig. 3A–3C). These results indicate that the Tanpo *GW5* allele is recessive and has an additive effect on both grain width and thickness. However, the grain length of F_2_ individuals homozygous for the Tanpo *GW5* allele was significantly shorter than that of heterozygotes and Akitakomachi homozygotes (Fig. 3D). A slightly shorter grain length was observed in heterozygous compared to the Akitakomachi homozygous, which suggests that the *GW5* allele from Tanpo contributes to the shorter grain length.

### Analysis of the Tanpo *GW5* gene

**Fig. 4.**
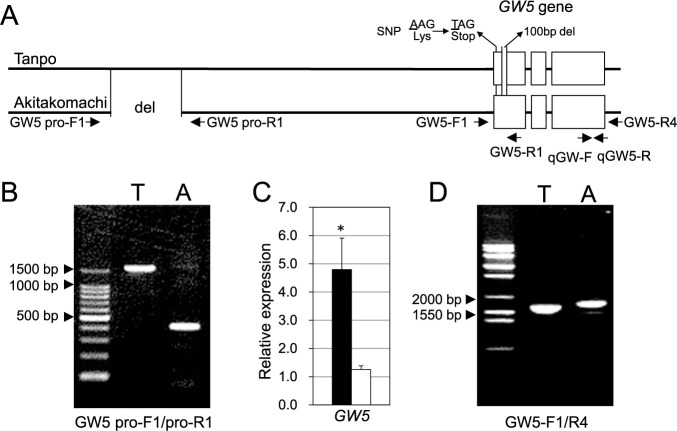
Analysis of the *GW5* gene in Tanpo and Akitakomachi. Panel A shows the *GW5* gene structure, including the promoter regions of Tanpo and Akitakomachi. Arrows indicate the primers used to analyze the gene structure. Panel B shows the amplified DNA fragments of the promoter regions of Tanpo (T) and Akitakomachi (A). Panel C shows a comparison of the relative expression of the *GW5* gene in the young inflorescences. White and black bars represent Akitakomachi and Tanpo, respectively. Values represent the mean ± standard error (SE) (n = 4). Single asterisks indicate significant differences between Tanpo and Akitakomachi as determined using the Student’s t-test (*P* < 0.05). Panel D shows the amplified DNA fragments of the entire *GW5* gene region from Tanpo (T) and Akitakomachi (A).

Previous studies have revealed that a 1212-bp deletion in the promoter region of the *GW5* gene in Japonica rice reduces *GW5* expression, thus resulting in wider grains (Liu *et al.* 2017). We analyzed the promoter region of the *GW5* gene in Tanpo and found that there was no deletion, which suggests a promoter structure similar to that of Indica rice (Supplemental Table 1, Fig. 4A, 4B). The *GW5* gene expression was significantly higher in the young panicles of Tanpo compared to Akitakomachi (Fig. 4C), indicating that the promoter region alone does not account for the characteristic grain shape of Tanpo.

The entire region of the *GW5* gene from Tanpo was shorter than that of Akitakomachi, which suggests that deletion had occurred within the Tanpo *GW5* gene (Fig. 4A, 4D). Further analysis revealed a 100-bp deletion within the first exon of the *GW5* gene in Tanpo. Moreover, a single nucleotide substitution generated a stop codon immediately after the start codon, thus resulting in a loss-of-function allele (Supplemental Fig. 3). GW5 protein deficiency in Tanpo likely contributes to the increased grain width and thickness observed compared to common Japonica varieties with reduced *GW5* expression.

### Expression profile of BR-related genes

GW5 has been shown to function as a positive regulator of BR signaling, thereby affecting grain width and weight (Liu *et al.* 2017). We analyzed the expression profiles of BR biosynthetic genes (*D2*, *DWARF*, and *CPD*) and BR signaling genes (*BRI1*, *GSK2*, *BZR1*, and *BU1*) in the young inflorescences of Tanpo and Akitakomachi cultivars.

**Fig. 5.**
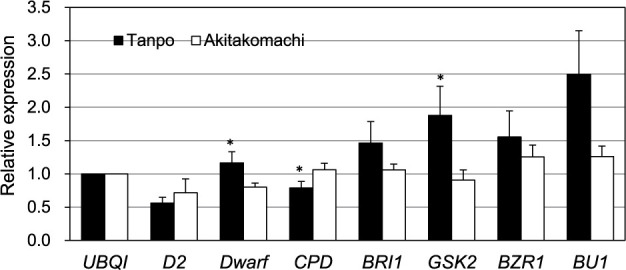
RT-qPCR analysis of genes related to brassinosteroid (BR) biosynthesis (*D2*, *Dwarf*, and *CPD*) and BR signaling (*BRI1*, *GSK2*, *BZR1*, and *BU1*) in the young inflorescences. Black and white bars represent Tanpo and Akitakomachi, respectively. Values represent the mean ± standard error (SE) (n = 4). Single asterisks indicate significant differences between Tanpo and Akitakomachi as determined using the Student’s *t*-test (*P < 0.05).

While the expression of the BR biosynthesis gene *CPD* was downregulated in Tanpo, the expression of another BR biosynthesis gene, *DWARF*, was upregulated (Fig. 5). Considering the multistep nature of BR biosynthesis, the loss of GW5 function may not affect the overall BR biosynthesis in the young inflorescences of Tanpo. However, we observed a significant upregulation of GSK2, a negative regulator of BR signaling, in Tanpo compared to Akitakomachi (Fig. 5). In addition, the expression of other BR signaling genes, *BRI1*, *BZR1*, and *BU1*, were also higher in Tanpo, which suggests that the loss of *GW5* function affected BR signaling in the young Tanpo inflorescences.

## Discussion

### Role of the *GW5* gene in determining Tanpo grain shape

The Japanese landrace rice Tanpo was widely cultivated in Akita Prefecture during the Meiji period and exhibits unique grain characteristics, including a shorter, wider, thicker, and heavier shape compared to modern cultivars. Microscopic analysis revealed that the epidermal cells of the Tanpo spikelet hulls were significantly smaller in both length and width than those of Akitakomachi. Because the spikelet hulls were wider in the Tanpo cultivar than in Akitakomachi, the number of cells in the grain width direction in the spikelet hulls was increased in Tanpo. The spikelet hull plays a predominant role in determining the grain size and shape as a container during grain filling (Li and Li 2016, Li *et al.* 2018). For instance, rice *sts-1* mutants exhibited short grains because of reductions in both cell length and cell number in the longitudinal direction of the spikelet hulls (Abe *et al.* 2010). Therefore, the distinctive grain shape of Tanpo is likely attributed to a combination of the reduced cell length and increased cell number in the grain width direction of the spikelet hulls.

The *GW5* gene, encoding a calmodulin-binding protein, was identified as a major QTL on chromosome 5 that regulates grain width and weight (Duan *et al.* 2017, Liu *et al.* 2017). The Tanpo *GW5* allele additively increased the grain width, thickness, and weight while decreasing the grain length in the F_2_ population derived from a cross between Tanpo and Akitakomachi. As the shape of the F_3_ grains was strongly influenced by the shape of the parental F_2_ spikelet hulls, the grain shape of the F_2_ individuals reflected their respective F_2_ genotypes. These results indicate that the Tanpo *GW5* allele is recessive and contributes to the shorter, wider, thicker, and heavier grain characteristics.

The Tanpo *GW5* gene has a loss of function because of a nonsense mutation and a 100-bp deletion within the first exon. Previous studies have shown that reduced *GW5* gene expression in Japonica varieties results in wider grains (Duan *et al.* 2017, Liu *et al.* 2017). The GW5 protein deficiency in Tanpo, resulting from the loss-of-function allele, likely contributes to the further increase in grain width and thickness compared to common Japonica varieties with reduced *GW5* expression.

GW5 protein physically interacts with and suppresses GSK2, a positive regulator of BR signaling (Liu *et al.* 2017). GSK2 restricts cell expansion in spikelet hulls, thus resulting in longer and wider grains (Che *et al.* 2016, Lyu *et al.* 2020, Zhang 2020). In Tanpo, the loss of *GW5* function prevents GSK2 suppression, which results in restricted epidermal cell expansion in spikelet hulls and smaller cells.

The overexpression of *GW5* has been shown to reduce the number of epidermal cells in the grain width direction on the spikelet hulls (Liu *et al.* 2017), while knockout of *GSE5* (*GW5*) results in narrower, more numerous epidermal cells (Duan *et al.* 2017). These findings suggest that *GW5* (*GSE5*) controls grain width by regulating cell proliferation in the spikelet hulls (Duan *et al.* 2017). In Tanpo, the loss of *GW5* function likely promotes the formation of shorter and broader spikelet hulls by increasing the cell number in the grain width direction while decreasing the cell size.

### BR signaling and grain shape in Tanpo

**Fig. 6.**
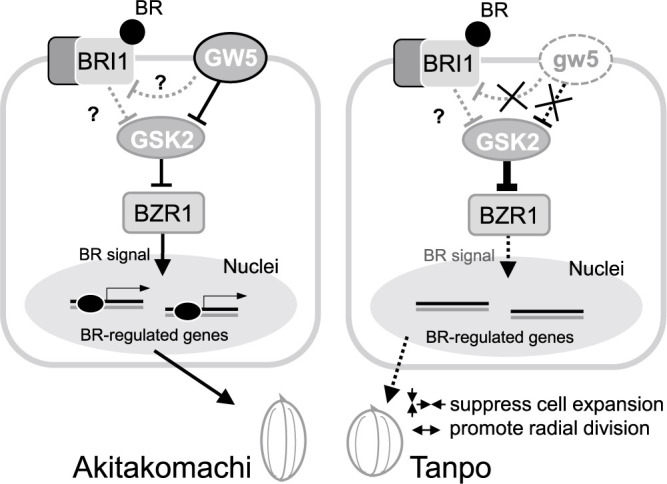
Proposed model for the regulation of grain shape in the Tanpo cultivar based on the model report by Liu *et al.* (2017). GW5 represses GSK2, which controls BZR1 via phosphorylation. BZR1 induces the expression of brassinosteroid (BR)-responsive genes, which leads to normal cell growth in the spikelet hulls of Akitakomachi. However, in Tanpo, GSK2 is not suppressed because of the absence of the GW5 protein, which strongly suppresses BR signaling and results in the suppression of cell expansion but enhances cell division in the grain width direction of the spikelet hulls.

Fig. 6 summarizes the regulation of grain shape by the Tanpo *GW5* allele via BR signaling, based on the model proposed by Liu *et al.* (2017). The GW5 protein is localized to the plasma membrane and suppresses the kinase activity of GSK2, a key component of BR signaling (Liu *et al.* 2017). GSK2 phosphorylates the transcription factor BZR1, inhibiting its accumulation in the nucleus and ultimately affecting BR-responsive gene expression (Tong *et al.* 2012, Zhang *et al.* 2014). In rice, GW5 acts positively and upstream of GSK2 in the BR signaling pathway (Liu *et al.* 2017). In Tanpo, GSK2 function was not suppressed by GW5 because of its loss of function. Moreover, GSK2 expression was upregulated, suggesting that BR signaling is suppressed in the Tanpo cultivar. To compensate for this, the expression of *BU1*, a BR receptor, and *BZR1* and *BRI1*, transcription factors for BR-responsive genes, may be upregulated in the young Tanpo inflorescences.BR plays a crucial role in regulating various agronomic traits in rice, including grain development (Chen *et al.* 2021, Xiong *et al.* 2022). For example, BR-deficient rice mutants, such as *ebisu dwarf* (*d2*), exhibit shortened grains (Hong *et al.* 2003). Therefore, the reduction in BR signaling in the young Tanpo inflorescences likely resulted in smaller cells while increasing cell proliferation in the grain width direction in the spikelet hulls (Fig. 6).

BR has been shown to suppress cytokinin responses, which induce the periclinal division of vascular cells in the *Arabidopsis* root apical meristem (Ohashi-Ito *et al.* 2023). The reduced BR signaling in Tanpo spikelet hulls may have led to a lack of cytokinin response suppression, potentially resulting in thicker grains via cytokinin-induced periclinal division within the grains.

### Breeding using the *gw5* allele of Tanpo

The non-functional *gw5* allele in Tanpo results in wider and thicker but shorter grains. Genome editing to create a loss-of-function *GSE5* (*GW5*) allele in an Indica variety has been shown to result in wider grains without reducing the grain length (Duan *et al.* 2017). This suggests that the genetic background influences the grain shape, as numerous genes are involved in regulating the grain size and shape (Chen *et al.* 2021, Li *et al.* 2022). The *gw5* allele, a natural variation found in the landrace Tanpo, could be used to breed varieties with larger and heavier grains by increasing the width and thickness, in addition to length.

Thicker rice grains in Koshihikari, a Japanese good-tasting variety, are known to have a higher amylose and lower protein content, which contributes to the improved taste (Kuroda and Matsumoto 1996). In addition, grain thickness has a positive effect on the taste quality (He *et al.* 2022). These findings suggest that the Tanpo *gw5* allele could also contribute to improving the taste quality by increasing the grain thickness.

Tanpo exhibits undesirable traits, such as excessive plant height (over 120 cm) and poor appearance quality of brown rice. Rice mutants with reduced brassinosteroid biosynthesis are known to exhibit decreased plant height (Hong *et al.* 2003), suggesting that the Tanpo *gw5* allele, which suppresses brassinosteroid signaling, does not contribute to increased plant height in Tanpo. Therefore, the *gw5* allele in Tanpo can be specifically utilized to enhance seed morphology. By integrating the Tanpo *gw5* allele into the genetic background of a leading variety with a superior brown rice quality through marker-assisted selection targeting the 100-bp deletion, Tanpo can serve as a valuable genetic resource. The *gw5* allele in Tanpo represents a novel natural mutation that promotes large grain size and holds potential for high-yielding breeding programs in the future.

## Author Contribution Statement

KS and HA designed the research. RN and SM developed the materials. MI, KC, and RN performed data collection and analysis. MI and KU performed microscopy analysis. MI and AW performed RT-qPCR analysis. MI, HA, and KS wrote the manuscript. All authors commented on the manuscript.

## Supplementary Material

Supplemental Figures

Supplemental Tables

## Figures and Tables

**Table 1.** Comparison of the grain shape of Tanpo and Akitakomachi

Variety	Grain length (mm)	Grain width (mm)	Grain thickness (mm)	Grain weight (mg)
Akitakomachi	5.38 ± 0.20**	2.77 ± 0.08**	2.07 ± 0.08**	23.0 ± 0.35**
Tanpo	4.86 ± 0.27	2.92 ± 0.11	2.21 ± 0.08	26.3 ± 0.59

Mean ± SD (n = 14) Double asterisks indicate significant difference at *P* < 0.01.
