# Supplementary material for: Loss of GW5 function is involved in the unique grain shape of “Tanpo”, a Japanese landrace rice
Source: Breed Sci. 2025 Mar 27;75(2):147–53. doi: 10.1270/jsbbs.24076 (PMC12395201; doi:10.1270/jsbbs.24076)
Supplement: Supplementary file 1 — Supplemental Figures [file 75_147_s1.pdf]

doi: 10.1270/jsbbs.24076

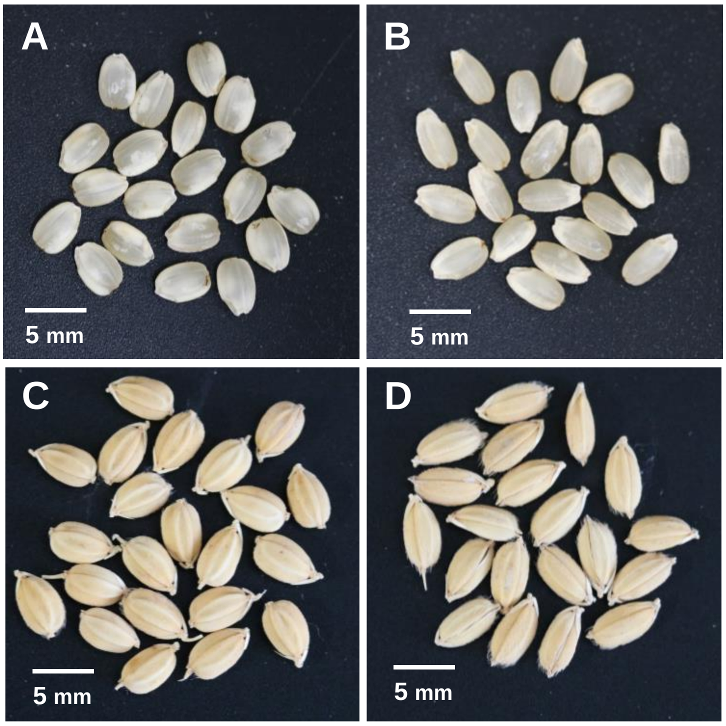

Supplemental Fig. 1 Comparison of grains between Tanpo and Akitakomachi. Brown rice and seeds from Tanpo (A and C) and Akitakomachi (B and D). Awns were removed from Tanpo seeds to clearly observe the shape of the seeds (C). Scale bar = 5 mm.

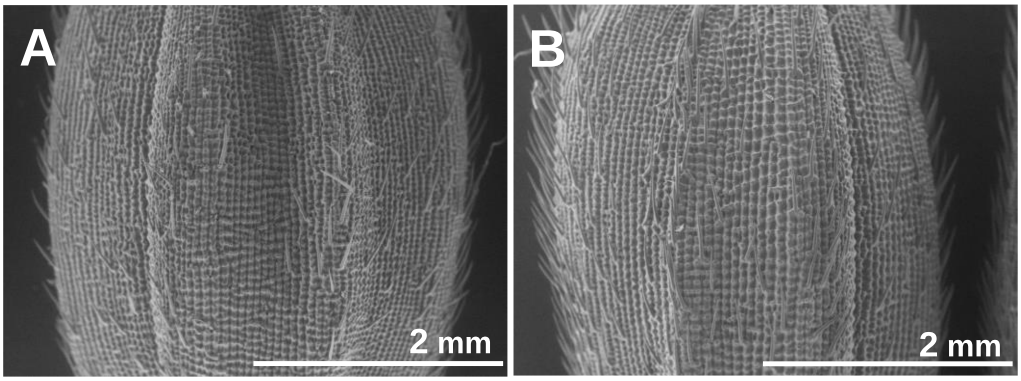

Supplemental Fig. 2 Scanning electron microscope analysis of the spikelet hulls from Tanpo (A) and Akitakomachi (B). Scale bar = 200 µm.

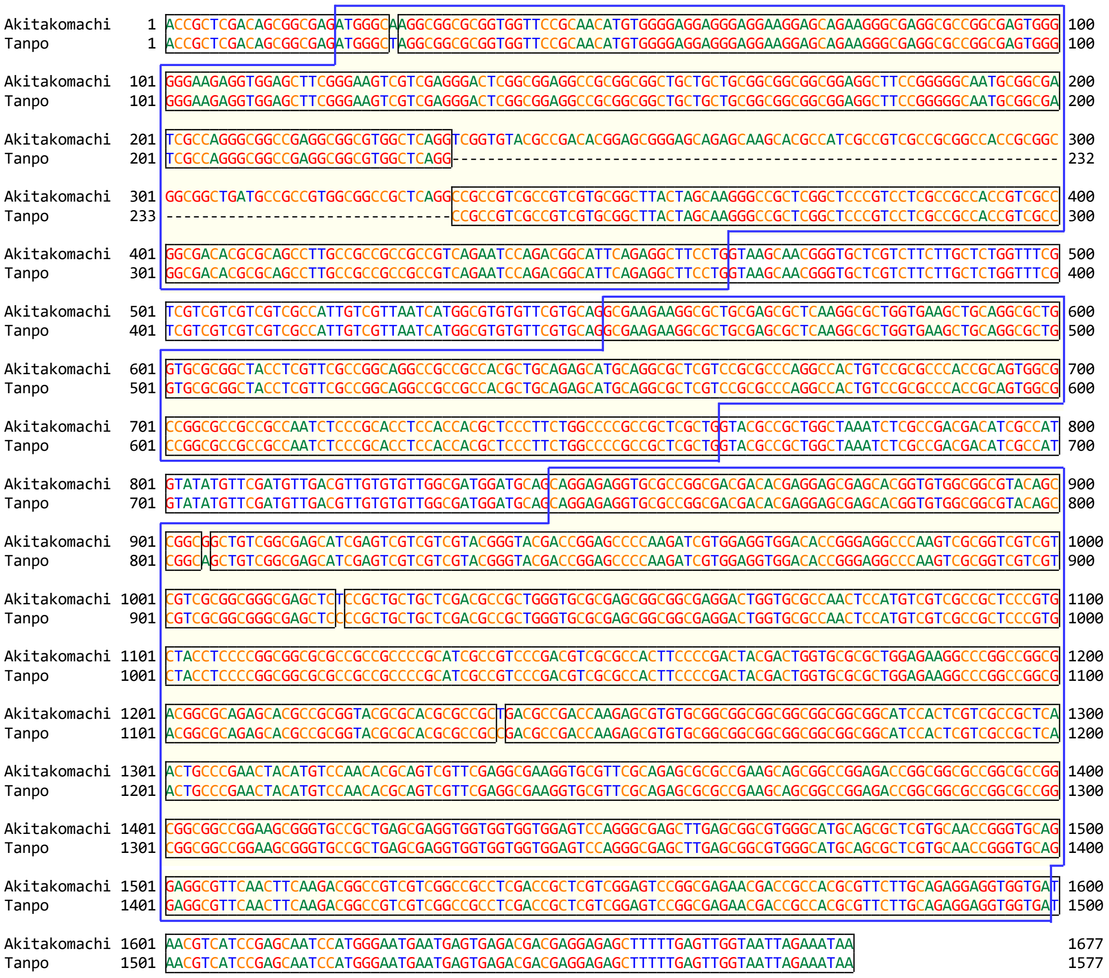

Supplemental Fig. 3 Comparison of the nucleotide sequence of the *GW5* gene between Tanpo and Akitakomachi. The three regions with yellow backgrounds represent the exons. There are four base substitutions and a 100-bp deletion in Tanpo. The base substitution immediately after the start codon generates a TAG stop codon.
