# Supplementary material for: Loss of GW5 function is involved in the unique grain shape of “Tanpo”, a Japanese landrace rice
Source: Breed Sci. 2025 Mar 27;75(2):147–53. doi: 10.1270/jsbbs.24076 (PMC12395201; doi:10.1270/jsbbs.24076)
Supplement: Supplementary file 2 — Supplemental Tables [file 75_147_s2.pdf]

doi: 10.1270/jsbbs.24076

Supplemental Table 1. PCR primer for analysis of *GW5* gene.

Gene	primer name	primer sequence	Tanpo	Akitakomachi
GW5 genotyping	GW5 F	GACTCCTCACCCGCTAGCTA	501 bp	601 bp
	GW5 R	AATGCCGTCTGGATTCTGAC		
GW5 gene	GW5 F1	GACTCCTCACCCGCTAGCTA	1648 bp	1748 bp
	GW5 R4	AGAGAGCTCCCAAGAACACA		

Supplemental Table 2. PCR primer for analysis of *GW5* promoter

gene	primer name	primer sequence	indica	japonica
GW5 promoter	GW5 pro F1	GTCCACACACCTGCAAGTTT	1609 bp	398 bp
	GW5 pro R1	TCTACTCCTAGTGAGGAAGG		

Supplemental Table 3. Primer sequences used for qPCR

Gene	primer sequence*
QUBI	GCTCCGTGGCGGTATCAT<br>CGGCAGTTGACAGCCCTAG
GW5	GCTTGAGCGGCGTGGGCAT (qGW5-F)<br>TCCTCTGCAAGAACGCGTGGC (qGW5-R)
D2	CCTTTTGGTGGTGGGCAGAG<br>TGGGGAAGTTGACGATGTGGT
DWARF	GAGGTGGCTGGAGAAGAACAT<br>ATTTCTACGGTGCCTACTTCCT
CPD	CAACAATAAACTTCAGAATGCGGT<br>GCGTTACAAGATGATGGAGGAAG
BRI1	GCAAGGGTATCTGATTTCGGT<br>CAAGAGTGGACACGCTAAGGT
GSK2	CAGTTATTTAGGGGGCTTGCGT<br>CTCGGTAGTAGCGTGAGCATA
BZR1	GCCGAGCAAAAAAGATGGTTC<br>AAATCGCCCAAATCGCAGCAT
BU1	CATCTCCAAGCTCCAGTCCCT<br>GCTCTTGATGTAGCTGCACGTCT

* Liu et al. 2017
